# Cryoglobulinemic glomerulonephritis with underlying occult HBV infection and Waldenström macroglobulinemia: A case report

**DOI:** 10.1097/MD.0000000000024792

**Published:** 2021-02-19

**Authors:** Yu-Che Chuang, Ying-Ren Chen, Te-Hui Kuo

**Affiliations:** a Education Center, National Cheng Kung University Hospital; b Department of Pathology, National Cheng Kung University Hospital; c Division of Nephrology, Department of Internal Medicine, National Cheng Kung University Hospital; d Department of Public Health, College of Medicine, National Cheng Kung University, Tainan, Taiwan.

**Keywords:** cryoglobulinemia, glomerulonephritis, hepatitis B surface antigen, non-Hodgkin lymphoma, occult HBV infection, Waldenström macroglobulinemia

## Abstract

**Introduction:**

Occult hepatitis B virus (HBV) infection, defined as negative hepatitis B surface antigen (HBsAg) but detectable HBV DNA in serum and liver tissue, has very rarely been described in cryoglobulinemia (CG) patients. This case report sheds light on the possible link between occult HBV infection and CG.

**Patient concerns:**

A 76-year-old man presented with rapidly deteriorating renal function within 1 year.

**Diagnosis:**

Cryoglobulinemic glomerulonephritis was diagnosed through renal biopsy. Initially, the patient tested negative for HBsAg, but a low HBV viral load was later discovered, indicating an occult HBV infection. Further studies also revealed Waldenström macroglobulinemia (WM).

**Interventions:**

We treated the patient as WM using plasma exchange and rituximab-based immunosuppressive therapy.

**Outcomes:**

After 1 cycle of immunosuppressive treatment, there was no improvement of renal function. Shortly after, treatment was discontinued due to an episode of life-threatening pneumonia. Hemodialysis was ultimately required.

**Conclusion:**

Future studies are needed to explore the link between occult HBV infection and CG, to investigate the mediating role of lymphomagenesis, and to examine the effectiveness of anti-HBV drugs in treating the group of CG patients with occult HBV infection. We encourage clinicians to incorporate HBV viral load testing into the evaluation panel for CG patients especially in HBV-endemic areas, and to test HBV viral load for essential CG patients in whom CG cannot be attributed to any primary disease.

## 1 Introduction

The current classification of cryoglobulinemia (CG) originated from findings published more than 40 years ago, in which cryoglobulins of 86 patients were grouped into 3 types based on immunochemical analysis.^[1]^ Most studies have extrapolated from this classification, further describing cryoglobulins as monoclonal immunoglobulins (Ig) in type I CG, monoclonal Ig with rheumatoid activity (usually IgM) plus polyclonal IgG in type II CG, and polyclonal IgM plus polyclonal IgG in type III CG.^[2]^ While current studies have identified lymphoproliferative diseases such as Waldenström macroglobulinemia (WM) as well as active hepatitis B virus (HBV) infection as the cause of CG, few, if any at all, have mentioned the role of occult HBV infection, defined as negative hepatitis B surface antigen (HBsAg) but detectable HBV DNA in serum and liver tissue,^[3]^ in CG. This case of cryoglobulinemic glomerulonephritis contributes to scarce literature on this topic and emphasizes the potential link between occult HBV infection and CG.

## 2 Case report

A 76-year-old man was referred to our unit due to progressive lower limb edema for 1 month. He had medical history of type 2 diabetes mellitus, hypertension, and hyperlipidemia. His latest glycated hemoglobin (HbA1c) level was 6.6%. Around 1 year earlier, the patient noted numbness and tingling over distal toes, which gradually progressed to his distal fingers and ankles. Non-itchy, non-tender hyperpigmented macules over bilateral legs also appeared. These symptoms caught little attention of the patient until lower limb edema developed 1 month ago. Since then, decreased urine output, fatigue and near-syncope episodes were reported.

**Figure 1:**
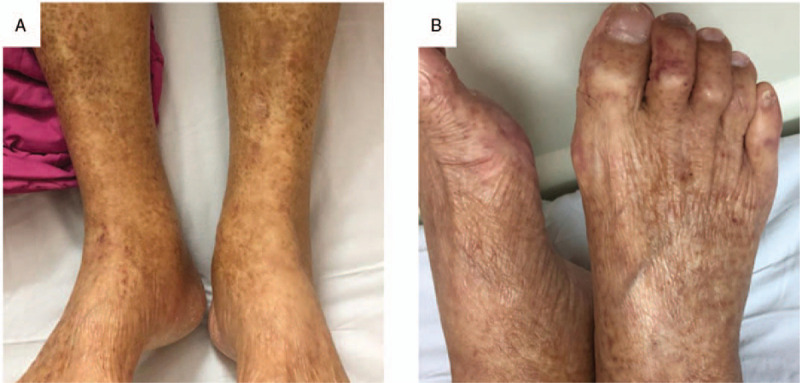
(a) Hyperpigmented macules with confluent patches over lower legs. (b) Petechiae over feet and toes.

Upon examination, we noted pitting edema over extremities, hyperpigmented macules with confluent patches over lower legs, and petechiae over both feet (Fig. 1a-b). Blood tests showed normocytic anemia (hemoglobin 9.9 g/dl), with normal white blood cell and platelet counts. Serum creatinine had risen from a basal level of 0.88 mg/dl to 2.13 mg/dl within 1 year, associated with microscopic hematuria (26–50/high power field), heavy proteinuria (urine protein to creatinine ratio 7643 mg/g), and hypoalbuminemia (3.2 mg/dl). His rapid deterioration of renal function was atypical for diabetic nephropathy, and renal biopsy was arranged. Immune studies prior to renal biopsy showed positive cryoglobulins in both serum and plasma, elevated rheumatoid factor (RF 883 IU/ml), normal complement component 3 (C3) (104.0 mg/dl), and reduced complement component (C4) (7.4 mg/dl), while antinuclear antibodies (ANA), anti-neutrophil cytoplasmic antibodies (ANCA), anti-double stranded DNA (anti-dsDNA) antibodies and anti-glomerular basement membrane antibodies were all negative. Infection screening showed negative results for HBsAg, hepatitis C antibodies (anti-HCV Ab), and Human immunodeficiency virus (HIV) antibodies.

**Figure 2:**
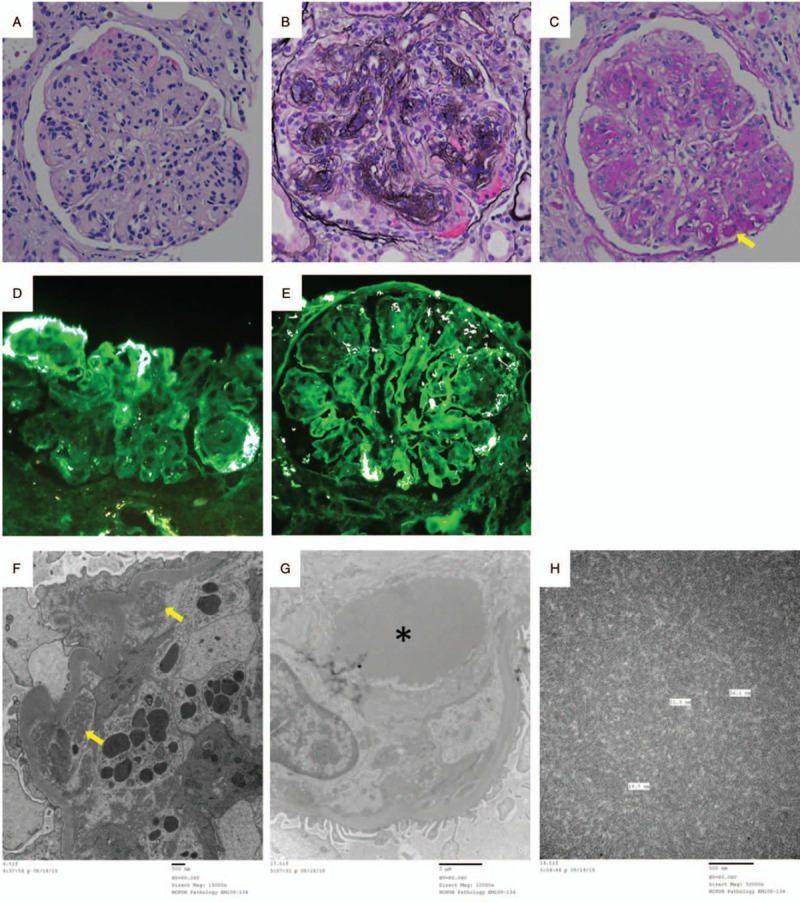
(A) H&E stain showing mesangial expansion and hypercellularity with prominent lobular accentuation (×400). (B) PAM stain highlighting double contours of capillary loops (×400). (C) PAS stain was positive for pseudothrombus (arrow) in the capillary (×400). (D) IgM immunofluorescence stain of a glomerulus showing segmental granular staining over capillary loops (×400). (E) IgG immunofluorescence stain also showing segmental granular staining over capillary loops (×400). (F) Electron microscopy revealing electron-dense deposits (arrows) in the subendothelial region (×15000). (G) Intraluminal thrombus (asterisk) (×12000). (H) Intraluminal thrombus composed of microtubular (10–30 nm wide) and ring-like structures (×50000).

Renal biopsy yielded 15 glomeruli, showing diffuse global endocapillary proliferation, mesangial expansion and hypercellularity with prominent lobular accentuation (Fig. 2a), diffuse thickening of glomerular basement membrane with focal segmental double contours of capillary loops on periodic acid-methenamine silver (PAM) stain (Fig. 2b), and eosinophilic, rounded periodic acid-Schiff (PAS) positive pseudothrombus in the capillary lumen (Fig. 2c). Immunofluorescence showed focal segmental coarse granular deposits of IgM (2+) (Fig. 2d), IgG (3+) (Fig. 2e), and C3 (2+) along the capillary walls. Electron microscopy revealed prominent circumferential cellular interposition composed of macrophages and mononuclear cells with electron-dense deposits in the subendothelial region (Fig. 2f), as well as intraluminal thrombi with organized deposits composed of microtubular (10–30 nm wide) and ring-like structures (Fig. 2g-h), supporting the diagnosis of cryoglobulinemic glomerulonephritis.

Subsequently, skin biopsy of petechial sites was done over his right ankle. In the upper dermis, small blood vessels were occluded by eosinophilic hyaline-like material, with extravasation of erythrocytes mixed with perivascular infiltration of neutrophils. Direct immunofluorescence showed positive stainings for IgM, IgG, and C3 over small blood vessels. These findings were compatible with cryoglobulinemic vasculitis.

Although qualitative testing of cryoglobulins could not be performed in our institution, the patient's symptoms were very likely caused by mixed cryoglobulins due to his elevated RF levels, reduced C4, and presence of both IgM and IgG staining on previous biopsies. Since no underlying infection or autoimmune disease was detected, the patient was first treated with oral prednisolone 55 mg/day for 1 month (patient weight 56 kg, height 163 cm). After 1 month of treatment, worsening renal function and persistent heavy proteinuria prompted further investigation. A thorough serological evaluation showed HBsAg negative, anti-HBs Ab weak positive, anti-HBc Ab positive, and an HBV viral load of 60 IU/ml was detected, indicating an occult HBV infection. HCV viral detection, anti-SSA and anti-SSB Ab were negative. Tests for hematological disorders including bone marrow biopsy were also sent. Since there was no evidence of active HBV or HCV infection, we treated the patient preemptively as essential mixed cryoglobulinemia. Oral prednisolone 55 mg/day was maintained and the patient received 1 dose of intravenous cyclophosphamide 500 mg, followed by oral cyclophosphamide 100 mg/day for 2 weeks.

**Figure 3:**
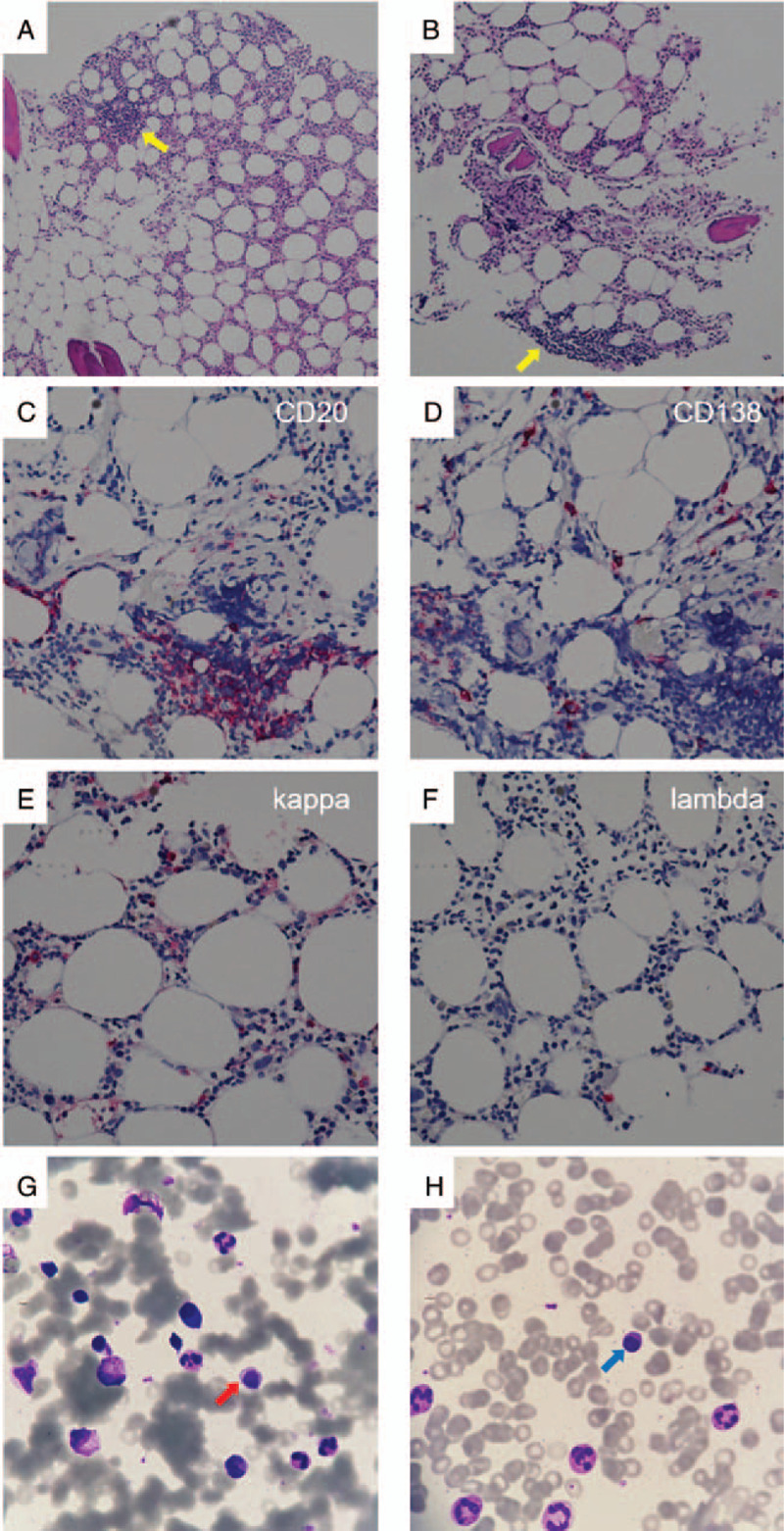
(A), (B) Bone marrow biopsy showing focal lymphoid aggregates (arrows) (×100). (C) CD20 showing abundant small B lymphocytes (×400). (D) CD138 highlighting plasma cells (×400). (E), (F) Plasma cells mostly kappa-positive and lambda-negative (×400). (G) Plasmacytoid lymphocytes (arrow) observed in bone marrow smear (Liu's stain) (×1000). (H) Plasmacytoid lymphocytes (arrow) observed in peripheral blood smear (Liu's stain) (×1000).

During this course of treatment, hematological tests revealed that his serum IgG was reduced (162 mg/dl) while IgM was increased (422 mg/dl); serum free kappa/lambda ratio was 5.19 (free kappa 94.4 mg/l, free lambda 18.18 mg/l). Serum protein electrophoresis and immunofixation showed IgM kappa monoclonal gammopathy, with a paraprotein of 2.9% (0.1 g/dl). Bone marrow biopsy showed focal lymphoid aggregates, composed of CD20 positive small B lymphocytes (Fig. 3a-c) which were negative for CD3, CD5, CD10, Myeloid cell nuclear differentiation antigen (MNDA), and cyclin D1 immunostains. CD138 immunostain highlighted few plasma cells, which counted 7%, with kappa light chain restriction (Fig. 3d-f). Plasmacytoid lymphocytes were observed in both bone marrow smears and peripheral blood smears (Fig. 3g-h). Due to these pathological findings, a diagnosis of WM was made. Chest and abdomen CT scans showed prominent mediastinal lymph nodes up to 2.0 cm, without hepatosplenomegaly. Further biopsy of the lymph nodes was not performed.

After establishing the diagnosis, we discontinued oral cyclophosphamide and tapered prednisolone in order to redirect treatment towards WM. The patient's follow-up serum creatinine levels a month later reached 3.82 mg/dl. He then received plasmapheresis for 5 times, followed by one dose of rituximab 600 mg, one dose of methylprednisolone 80 mg, and 5 days of oral cyclophosphamide 200 mg/day. Prophylactic entecavir was given prior to immunosuppressive therapy and lasted for 2 weeks. Two weeks after his first cycle of immunosuppressive regimen, no improvement of his renal function was observed (serum creatinine 4.19 mg/dl). Shortly after, he had an episode of life-threatening pneumonia and was unable to proceed with the second cycle. After recovery from that infectious episode, the patient was informed of the benefits and the risks of further immunosuppressive treatments. He later opted for observation and conservative management. During follow-up, his serum creatinine rose steadily. Hemodialysis was eventually started 12 months after lower limb edema and decreased renal function first appeared.

## 3 Discussion and conclusions

WM appearing in type II/III CG (mixed CG) is uncommonly reported. In a French cohort study conducted by Kolopp-Sarda et al, only 1 out of 1520 type II/III CG (mixed CG) cases was associated with WM.^[4]^ However, it was unclear if all mixed CG patients in that study routinely received bone marrow studies, so an underestimation might be possible. One may reasonably wonder if bone marrow studies were even necessary once an underlying infection was found.

Many mixed CG cases deemed idiopathic (“essential”) in the early years, were found related to HCV after its discovery in 1989.^[2]^ Other mixed CG cases that were originally thought to be caused by primary lymphoproliferative diseases have also been put into question, due to evidence that these lymphoproliferative diseases may have been itself secondary to HCV infection. The evidence includes epidemiologic studies, one of which demonstrated a 20% to 30% increased risk of non-Hodgkin lymphoma (NHL) and a 3-fold higher risk of WM in HCV-infected American veterans.^[5]^ Several hypotheses for HCV-related lymphomagenesis have also been put forward,^[6]^ including continuous antigen stimulation of certain B-cell clones, and direct infection of B cells leading to transformation.

Apart from HCV, hepatitis B virus (HBV) infection has also emerged as one of the causes of mixed CGs, appearing in 5 out of 133 non-HCV-related mixed CG cases in a retrospective study.^[7]^ It is important to mention, however, that most studies evaluating HBV-related mixed CG used positive HBsAg as the marker for HBV infection. Occult HBV infection was often not accounted for. As a result, the association between occult HBV infection and CG remains unclear.^[8]^ Some essential mixed CG cases might actually have occult HBV infection if HBV viral load detection and HBsAg screen were ordered together. In this regard, we found that our case was strikingly similar to a case of cryoglobulinemic glomerulonephritis reported by Vázquez et al.^[9]^ Their case had underlying occult HBV infection and IgM kappa gammopathy. WM was later diagnosed through bone marrow studies. The authors recognized WM as the cause of CG, and the patient recovered after rituximab treatment. Interestingly, prophylactic tenofovir was given in advance to prevent HBV reactivation, which raises the question of whether tenofovir contributed partly to that patient's recovery through suppressing HBV replication. Before our patient's first cycle of immunosuppressive regimen, entecavir was given as prophylaxis, but was discontinued 2 weeks later. Based on some reports of HBV reactivation in occult HBV patients after receiving chemotherapy and immunosuppressive therapy, Lledó et al recommended a prudent approach of continuing antiviral therapy for more than 6 months after immunosuppressive treatment had been stopped.^[10]^ We are aware of this recommendation and have been monitoring the patient closely for any signs of viral reactivation.

Another question raised is whether occult HBV infection in our case had a causal relationship with WM. The proposed mechanisms of HBV lymphomagenesis have been somewhat similar to that proposed for HCV lymphomagenesis, including chronic surface antigen stimulation^[11]^ and malignant transformation of B-cells through oncogenic signals.^[12]^ A meta-analysis of 22 studies^[13]^ found that the odds of developing NHL increased by 2.24 times in HBV-infected patients, and that after subtype analysis, there was a statistically significant risk of developing diffuse large B-cell lymphoma, follicular lymphoma, or T-cell lymphoma. Again, despite epidemiological studies demonstrating the connection between active HBV infection and NHL, occult HBV infection cases were mostly unaccounted for. Notably, a Taiwanese study found a higher prevalence of occult HBV infection in NHL patients (6%) compared to solid tumor patients (0%) and healthy controls (0.9%).^[14]^ However, none of those NHL cases with occult HBV belonged to the lymphoplasmacytic lymphoma/WM subtype.

In summary, the contribution of occult HBV infection to the pathogenesis of CG, which is potentially mediated by lymphomagenesis, may have been under-recognized due to the widespread screening method using only HBsAg. If future reports emerge that cryoglobulinemic symptoms in occult HBV patients can be successfully treated with anti-HBV drugs alone, this will prove the causal relationship between occult HBV infection and CG, thereby establishing the necessity of testing HBV viral load in CG patients who had originally tested negative for HBsAg.

## 4 Availability of data and materials

All data generated or analyzed during this study are included in this published article.

## Author contributions

YCC collected data and wrote the first draft; YRC was responsible for pathological diagnosis and revised pathological descriptions in the manuscript; THK was responsible for clinical care of this patient, reviewed the final version and made the final edits. All authors have read and approved the manuscript.

**Writing – original draft:** Yu-Che Chuang.

**Writing – review & editing:** Te-Hui Kuo, Ying-Ren Chen.
